# Pedestrians’ adherence to road traffic regulations on the N1 Highway in Accra, Ghana

**DOI:** 10.11604/pamj.supp.2016.25.1.6184

**Published:** 2016-10-01

**Authors:** Charles Lwanga Noora, Edwin Andrews Afari, Robert Domo Nuoh, Eric Yirenkyi Adjei, Gershon Kobla Anthony, Marijanatu Abdulai, Samuel Oko Sackey, Ernest Kenu, Kofi Mensah Nyarko

**Affiliations:** 1 School Of Public Health, University of Ghana, Legon Boundary, Accra, Ghana; 2 Ghana Field Epidemiology And Laboratory Training Program; 3 Ghana Health Service, Ghana

**Keywords:** Highway, pedestrian, unapproved route, road traffic accidents, footbridge, Ghana

## Abstract

**Introduction:**

Pedestrian behavior and adherence to road traffic regulation is vital in the prevention and control of road traffic accidents (RTA) especially on highways in Ghana. We assessed pedestrians’ adherence to road crossing regulations on the George Walker Bush (N1) Highway in Accra.

**Methods:**

We conducted a cross sectional study of pedestrians crossing the N1 highway from both sides of the road between 7:00 am and 11:00am. We observed all pedestrians using a checklist and interviewed 413 using a structured questionnaire. We collected data on basic demographics, and pedestrians’ knowledge on road crossing (exposures). Data was, cleaned and analyzed using Epi-info version 3.5.4. Pearson Chi-square was used to assess differences in proportions for categorical variables. Binary logistic regression was used to test for association between pedestrian choice of route and exposures.

**Results:**

We observed (n = 1856) pedestrians crossing the road during the study period; 1155 (62.2%) males, 461 (24.8%) did not use the approved route(s). Majority 317(76.8%) were adults between the ages of 20-49, mostly males 265 (56.4%). Most people (92.7%) had at least basic education. AOR for sex (male) was 1.7(1.1-2.6), and regular use of Highway (always) was 0.4(0.2-0.8) at 95% CI.

**Conclusion:**

One out of every 4pedestrians using the N1 Highway used an unapproved route. Majority of pedestrians who regularly cross the Highway at unapproved routes were males. We recommend vigorous public education and addition of more footbridges.

## Introduction

Globally an estimated 270,000 pedestrians die on our roads annually representing 22%of all road traffic accidents [1]. The global risk of dying through road traffic accidents in US, is about 15 per 100,000 populations [2] while in Africa is estimated as 24.1 per 100,000 population [3]. Road traffic accidents are common in Ghana and more than 2000 lives are lost annually through road traffic accidents. Evidence show that, between 45-55% of these accidents involve pedestrians [4, 5]. Pedestrian crashes are widely known to be more common among males in all regions and across all age groups [6, 7]. Children below 15 years and the elderly above 70 years are mostly victims [8]. Studies have shown that single vehicle crashes in Botswana, Zimbabwe and Ghana involve pedestrians [9, 10]. Pedestrian behavior and adherence to road traffic regulations could predispose them to pedestrian crashes. In Ghana, 61.5% of pedestrian Road traffic fatalities have been recorded between 2001 and 2012 [10], and accounts for 43% of all road traffic accidents in Ghana [10].

In Ghana, road safety remains a serious public health challenge as the safety of all categories of road users is a major concern to all key stakeholders [9]. In recent times there have been frequent accidents involving pedestrians on the newly opened N1 Highway [11]. After 11 months of inauguration of the N1 Highway, crash statistics indicates that389 Road Traffic Crashes occurred on the N1, between the period February 14, 2012 and December 31, 2012, registering 52 fatalities and 284 injuries. A further, 112 pedestrians were run over by motor vehicles within the same period [8, 12]. Thus at least one person died and six others got injured every week through accidents on the Highway within the period.

Several factors have been attributed to road accidents in Ghana. Some of these include lack of pedestrian (Zebra) crossings, inadequate road signs, few or no footbridges, drunk driving, and lack of knowledge on road traffic regulations. Despite efforts by the National Road Safety Commission, the Police and the media to promote, co-ordinate and develop national road safety strategies to reduce or eliminate road fatalities, pedestrian road traffic accidents are still on the increase [14]. A lot remains to be done on the part of pedestrians and other road users. Down the streets in Accra, it is easy to see pedestrians crossing the road, with little consideration for their safety. The worrying pedestrian death tolls are attributable to a number of factors including pedestrians’ behavior and knowledge on road safety regulation especially concerning Highway crossings while driver related factors such as drunk driving, over speeding, non maintenance of vehicles are thought to be contributing to the situation [10, 13]. Pedestrian safety is dependent mainly on people taking responsible decisions while crossing the road as well as the need for drivers to be careful and considerate [13]. We assessed pedestrians’ adherence to road crossing regulations on the George Walker Bush (N1) Highway in Accra.

## Methods

### Study design and location

We conducted a cross sectional study among pedestrians using the newly constructed 14 km long N1 Highway in Accra, Ghana’s capital. The Highway now boasts of a six-lane divided thoroughfare with four overpasses, one elevated circle interchange, street lighting, drainage, bus stops, extra-wide sidewalks with graded ramps, pedestrian walkovers, and stoplights at all major intersections [14]. Though, the refurbished Highway has enhanced mobility in the city. Pedestrian’s adherence to road traffic regulations was defined as one who crosses the Highway from an approved route including using a footbridge, traffic lights, or “zebra crossings”. Route of pedestrian was categorized for this study as approved and unapproved routes. A section of these pedestrians were interviewed. One out of six (6) footbridges on the N1 Highway was selected for the study. We defined far away as distance of at least 150 meters away from the footbridge.

### Sample size determination

To detect differences in proportions between pedestrians adhering to road traffic regulations with pedestrians not adhering to road traffic regulations. We used an assumed prevalence of pedestrians adhering to road traffic regulations of 50%, precision of 5%and confidence interval of 95% to calculate the sample size of 384 as minimum number of pedestrians to be interviewed.

### Inclusion criteria

All pedestrians using the N1 highway within a 200 meters radius at the selected pedestrian footbridge between 7: 00 hrs and 11: 00 hrs either via approved or unapproved route were included.

### Exclusion criteria

Persons with physical disability and children less than 10 years who were assisted to cross the road were excluded from the study because we wanted to avoid persons who are physically challenged or could not willingly decide for themselves.

### Data collection

Data was collected through interviews using a structured questionnaire and observation using a checklist. We counted all pedestrians crossing the Highway from either side during morning rush hours, between the hours of 7:00hrs and 11:00 hrs. Every fifth pedestrian was approached and consenting individuals interviewed, data were collected using pretested, standardized, interviewer administered structured questionnaires and a checklist. The interviews were conducted by Ghana Field Epidemiology and Laboratory Training Program (GFELTP) residents and the questionnaire was in English with a local language (Twi) translation. Information on basic demography, level of education, and pedestrians’ knowledge on road crossing were collected.

### Data analysis

Data was cleaned and analyzed using Epi-info version 3.5.4. Pearson Chi-square was used to assess differences in proportions for categorical variables, with p < 0.05 considered statistically significant. Binary logistic regression was used in a bi-variate analysis to test for association between choice of route by pedestrian and exposure variables at 95% Confidence interval (CI). Crude and adjusted odds ratios were calculated.

### Ethical concerns

We obtained permission from the department of NRSC and verbal consent sought from pedestrians. Confidentiality of the information gathered and anonymity of pedestrians were ensured.

## Results

The investigators observed a total 1856 pedestrians crossing the high way. Out of this, 461 (24.8%) of these pedestrians did not use the approved route. We observed broken fences in the median on the Highway which allowed pedestrians to easily cross. Meanwhile, the footbridges environment studied were generally clean, tidy with adequate street lightening system. We also interviewed 413 of the pedestrians, 180 (43.0%) of them did not use the approved route. Majority 261 (63.2%) were males. Most (56.4%) pedestrians were between 20 and 29 years. Of the413 pedestrians interviewed, majority 156 (88.6%) of the pedestrians crossing the Highway from an unapproved route did not feel safe compared with 22 (9.4%) of pedestrians using the footbridge who did not feel safe (Table 1). The study found 208/413 (57.0%) pedestrians using the right route but said it was not a crime or anyone to cross the highway from unapproved route.

**Table 1:** Demographic characteristics of pedestrians crossing the N1 Highway, Accra

Characteristics	Number of pedestrian	approved route n (233)	Unapproved route n (180)	Pearson Chi2 test
	N	No. (%)	No. (%)	
**Total counted**	1868	1395(75.2)	461(24.8)	
**Interviewed**	n= 413(%)	233(56.4)	180(43.6)	
**Agecat**				**0.028**
10-19	56(13.6)	32(57.1)	24(42.9)	
20-29	141(34.1)	94(66.7)	47(33.3)	
30-39	109(26.4)	57(52.3)	52(47.7)	
40-49	67(16.2)	31(46.3)	36(53.7)	
≥50	40(9.7)	19(47.5)	21(52.5)	
**Sex**				**0.021**
Female	152	97(63.8)	55(36.2)	
Male	261	136(52.1)	125(47.9)	
**Educational status**				0.210
No education	30	13(43.3)	17(56.7)	
Primary level	111	62(55.9)	49(44.1)	
Secondary	170	93(54.7)	77(45.3)	
Tertiary	102	65(63.7)	37(36.3)	
**Occupation**				0.145
Employed	285	154(54.0)	131(46.0)	
Unemployed	128	79(61.7)	49(38.3)	
**Frequency of Route**				**0.009**
Once	51	21(41.2)	30(58.8)	
Always	238	148(62.2)	90(37.8)	
Occasionally	123	63(51.3)	60(48.8)	
**It is unlawful using an unapproved route**				0.298
Yes		17(50)	17(50)	
No		208(57.8)	152(42.2)	
Not sure		8(42.1)	11(57.9)	

**Figure 1:**
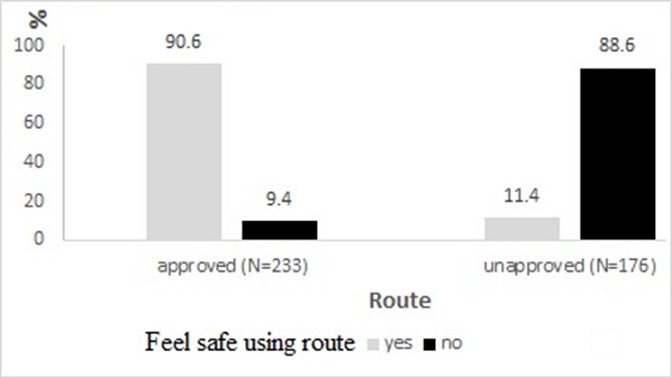
Safety of pedestrians by choice of route, N1 Highway, Accra

**Figure 2:**
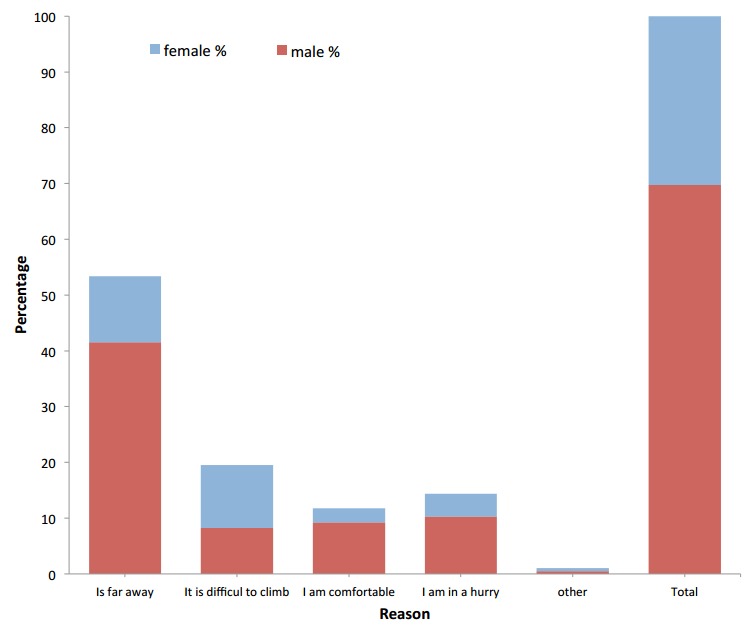
Pedestrians reason for choices of an unapproved route, N1 Highway, Accra

Of the two routes taken by pedestrians, 211 (90.6%) said they were safe crossing the Highway using the approved route while majority 156 (88.6%) of the pedestrians crossing the Highway from an unapproved route did not feel safe (Figure 1). Among the reasons for choosing to use an unapproved route, majority 94 (53.3%) of the pedestrians cited far distance from the footbridge and difficulty to climb footbridge. Only about 25 (14.4%) were in a hurry while some 20 (11.4%) were comfortable crossing the road from an unapproved route (Figure 2).

There were significant associations between age categories, sex, the usage of the various routes by pedestrians, and the route chosen by the pedestrian in a bi-variate analysis. However, only sex (male) with OR of 1.7 CI (1.1-2.6) and frequency of route usage; once with OR of 2.3 CI (1.3-4.4) and occasionally OR 1.6 (1.1-2.5) respectively showed significant association with p values < 0.05 in a logistics regression model (Table 2).

**Table 2:** Crude and modeled odds ratio of pedestrian’s characteristic on the N1 Highway, Accra-Ghana, 24-09-14

	Pedestrians using unapproved route n = 180, total pedestrians N=413				
Characteristics	% of n	Odds ratio (OR)	95%Confidence interval (CI)	Adjusted Odds ratio	95%Confidence interval (CI)
**Age category**					
10-19	42.9	1.0	Reference	1.0	Reference
20-29	33.3	0.7	0.4-1.3	0.6	0.3-1.1
30-39	47.7	1.2	0.6-2.3	1.1	0.6-2.1
40-49	53.7	1.5	0.8-3.1	1.4	0.7-2.9
≥50	52.5	1.5	0.6-3.3	1.3	0.6-3.0
**Sex**					
Female	36.2	1.0	Reference	1.0	Reference
Male	47.9	1.6	1.1-2.4	1.7	1.1-2.6
**Frequency of usage**					
Always	37.8	1.0	Reference	1.0	Reference
Once	58.8	2.3	1.3-4.4	2.4	1.3-4.5
Occasionally	48.8	1.6	1.0-2.4	1.6	1.1-2.5

## Discussion

Pedestrians, just like all other road users are supposed to obey all road signs and regulations especially rules on crossing Highways to ensure they use the road safely. Considering the number of pedestrian using the Highway within 4 hours of a day and within a diameter of 150 meters, the route can be said to be a busy Highway. This could be explained by the nature of the route; the Highway is constructed across several towns within the capital city of Ghana, Accra. The towns along the entire stretch of the road have residencies and business facilities on either sides of the Highway. This means that, residents who have businesses or have to transact business or access any social facility across the Highway may have to climb the pedestrian footbridge a number of times a day depending on the need and this could be exhausting. Majority (75%) of the pedestrians crossing the N1 Highway complied with road crossing regulations, this agrees with a similar study by Sisiopiku PV et al in the USA on Pedestrian behaviors at and perceptions towards various pedestrian facilities: an examination based on observation and survey data in which they found over 70% compliance rate [13]. Pedestrians crossing from unapproved route which directly put them at risk of car knockdowns were mostly young adults between the ages of 20 and 29 years but predominantly males. This could be due to the fact that males are more active than female [15]. This is consistent with a study by Amo T et al and Holland C et al which suggested that women aged 25-59 are less likely to cross Highways in risky situations than males [16]. This puts them at a greater risk of being knocked down by a car compared to pedestrians using the footbridge.

Most people had at least basic education; thus at the primary level (92.7%) as opposed to those with no education at all (7.3 %). The age group characteristics of pedestrians using the Highway either via the approved or unapproved route were similar, the study showed that, 77.7% were adults (20-49) years, 13% were teenagers (<20) years and 10%were, elderly (>50) years persons crossing the road from an unapproved route respectively while those on the an approved route, 78.1% were adults (20-49) years, 13.7% were teenagers (<20) years and 8.2%were, elderly (>50) years respectively.

Considering the response rate of pedestrians using an unapproved route, it is possible that that there are genuine concerns on the long distances between footbridges, thus providing more footbridges could potentially reduced over 40% of pedestrians crossing the Highway from an unapproved route. Also the fact that about 20% of pedestrian had difficulty in climbing the footbridge may be due to the fact that some of the people generally fear heights or have medical conditions that may not allow them to climb the footbridges. Interestingly over 15% of pedestrians crossing the Highway from an unapproved route were comfortable and a further 10% were in a hurry to cross the Highway. It is very possible that these pedestrians may be ignorant about the consequences or do not know the road crossing regulations.

We found more than 70% of pedestrians who clearly understood road safety crossing regulations, however almost 25% of pedestrians who live or work around the Highway still cross the road at unapproved points. There were significantly differences between frequency of road crossing by pedestrian and the choice of route taken by pedestrian (p=0.009), about 90% of pedestrians who used unapproved route did so always (p=0.009) as shown in Table 1 This could suggest that, the use of approved and unapproved routes is inherent in the people’s behavior. More than 80% (p=0.001) of pedestrians who use unapproved route agreed that it was not safe and had adequate understanding of road crossing regulations. Also, it was revealing to note that, majority of pedestrians crossing the highway from an approve route did not find anything wrong with others crossing from un approved routes. This probably is due to pedestrians’ luck of knowledge about road crossing regulations.

There was no significant association between age category and road crossing behavior in a modeled analysis but odds ratios suggest the choice of using an unapproved route to cross the road increased with increased in some age categories (Table 2). This is probably because older people generally find it difficult to climb the footbridge or older people feel they are careful enough in crossing the Highway from an unapproved route whiles young people may find it fun and exciting. There were significant association between sex and pedestrian crossing from an unapproved route in a multiple logistic regression model. The odds of being a male pedestrian and crossing the highway from an unapproved route is increased by 1.7 times compared to female pedestrian. The frequency of road usage was also found to be significantly associated with unapproved route. Comparing pedestrians who always use the highway to those who either occasionally or were crossing for the first time; the odds of a pedestrian occasionally and for the first time crossing the highway from an unapproved routes are increased by 1.6 and 2.4 times compared to pedestrians who always use the highway. This may be explained by the fact that, pedestrians who always use the Highway probable live around the Highway and might have suffered, seen or heard about the frequency of RTAs on the N1 Highway and therefore were more cautious than pedestrians either crossing the Highway for the first time or occasionally.

## Conclusion

On the basics of our findings, it was evident that the footbridge location, relative to the origin and destination of the pedestrian, was the most influential reason for pedestrians deciding to cross at a unapproved location (over 80% said so). About 25% of pedestrians on our Highway are directly exposed to pedestrian knockdown. Over 80% of these pedestrians did not feel crossing the Highway indiscriminately. We conclude that, indiscriminate crossing of the N1 Highway posses a significant public health threat to pedestrians on our Highways. The act of using approved or unapproved route in crossing the Highway was largely due to pedestrian behavior and attitude. More so, more than 20% of them found the footbridge difficult to climb and would rather cross just under the footbridge through openings. Therefore, Proper traffic control can further encourage pedestrian crossings at designated locations since the effect of the availability of pedestrian footbridge to influence pedestrians’ decisions to cross at an approved location was quite high. We recommend that; public education should be done to encourage the use of pedestrian footbridges; all unapproved routes in the median should be sealed while footbridges should be made more user friendly not only with staircase but also with a whirl round. In the long term, flyovers or under passes should be constructed by the ministry of roads and transport. There is need for further studies on pedestrian willingness, understanding and choice of road crossing route on major Highways in Ghana to fully understand any unmet need of pedestrians crossing the Highway.
